# Clinical applications of gene-based risk prediction for lung cancer and the central role of chronic obstructive pulmonary disease

**DOI:** 10.3389/fgene.2012.00210

**Published:** 2012-10-16

**Authors:** R. P. Young, R. J. Hopkins, G. D. Gamble

**Affiliations:** Faculty of Medical and Health Sciences, and Biological Sciences, University of AucklandAuckland, New Zealand

**Keywords:** lung cancer, COPD, risk prediction, genetic susceptibility, screening

## Abstract

Lung cancer is the leading cause of cancer death worldwide and nearly 90% of cases are attributable to smoking. Quitting smoking and early diagnosis of lung cancer, through computed tomographic screening, are the only ways to reduce mortality from lung cancer. Recent epidemiological studies show that risk prediction for lung cancer is optimized by using multivariate risk models that include age, smoking exposure, history of chronic obstructive pulmonary disease (COPD), family history of lung cancer, and body mass index. It has also been shown that COPD predates lung cancer in 65–70% of cases, conferring a four- to sixfold greater risk of lung cancer compared to smokers with normal lung function. Genome-wide association studies of smokers have identified a number of genetic variants associated with COPD or lung cancer. In a case–control study, where smokers with normal lungs were compared to smokers who had spirometry-defined COPD or histology confirmed lung cancer, several of these variants were shown to overlap, conferring the same susceptibility or protective effects on both COPD and lung cancer (independent of COPD status). In this perspective article, we show how combining clinical data with genetic variants can help identify heavy smokers at the greatest risk of lung cancer. Using this approach, we found that gene-based risk testing helped engage smokers in risk mitigating activities like quitting smoking and undertaking lung cancer screening. We suggest that such an approach could facilitate the targeted selection of smokers for cost-effective life-saving interventions.

## EPIDEMIOLOGY OF COPD

**FIGURE 1:**
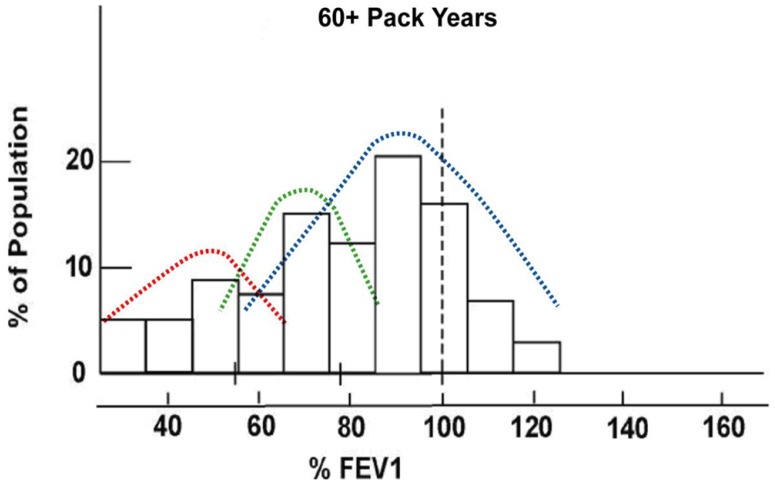
**Trimodal distribution of predicted FEV_**1**_ in current and former smokers with heavy smoke exposure defining “susceptible” and “resistant” smokers (modified from Burrows et al., 1977)**.

Chronic obstructive pulmonary disease (COPD) is characterized by fixed airflow obstruction, measured by spirometry as a reduced forced expiratory volume in 1 s (FEV_1_; Mannino et al., 2006). Based on both cross-sectional and prospective studies, it is estimated that 20–30% of smokers develop significant COPD while the remainder maintain near normal lung function (Løkke et al., 2006; Mannino et al., 2006; Kohansal et al., 2009). When smokers are stratified by smoking exposure dose, the distribution of %predicted FEV_1_ shifts from unimodal in light smokers to a trimodal distribution in heavy smokers (**Figure 1**; Young et al., 2007). This shift to trimodal distribution, following chronic smoke exposure, defines susceptible smokers with COPD and healthy smokers who are resistant to the effects of smoking. This observation shows that after three to four decades of heavy smoking exposure, a differential response to smoking can be observed that is independent of smoking exposure dose (Young et al., 2007). Such a discordant response to smoking exposure is consistent with a “pharmacogenetic effect” where variation in genetic makeup determines a person’s response to smoking exposure (i.e., drug exposure). These observations argue strongly in favor of a significant gene-by-environment effect whereby COPD occurs in smokers who are genetically susceptible and heavily exposed to decades of smoking (Molfino, 2004; Young and Hopkins, 2011d). Such an observation is unique in common complex diseases by defining two distinct phenotypes – susceptible (responder) phenotype (smokers with COPD) and resistant (non-responder) phenotype (smokers with normal lung function; Young et al., 2007).

Although 80–90% of those diagnosed with COPD have significant smoking exposure, there remains a group of 10–20% that have either been chronic asthmatics or people chronically exposed to other aero-pollutants (occupational dusts, passive tobacco smoke, or smoke from domestic cooking; Mannino et al., 2006). Irrespective of exposure, it is generally accepted that COPD results from the combined effects of a chronic airway inflammatory stimulus and host genetic susceptibility (Molfino, 2004; Young and Hopkins, 2011d). Twin studies have suggested that genetic factors underlie variation in airflow obstruction with an estimated heritability of 40–75% (Chen, 1999). This makes lung function a strong genetic trait in comparison with many other diseases or physiological states. While we conclude from these studies that reduced FEV_1_ (or reduced %predicted FEV_1_), characterizing COPD, is under significant genetic influence, smoking is an environmental exposure critical to the development of most COPD (and the penetrance of relevant “COPD” genes). As the relationship between smoking exposure and FEV_1_ may not be simply linear (Castaldi et al., 2011), simple adjustment by regression analyses may not be appropriate in case–control studies poorly matched for smoking history. We believe that much of the failure to replicate genetic associations in complex disease is due to small sample size and important differences in study design – in particular variation in defining the disease and control phenotype and, failure to allow for the effect of variable age and environmental exposure on expression of the phenotype (i.e., penetrance; Silverman et al., 2011). We suggest that COPD is an excellent example of a complex disease because the susceptible and non-susceptible (resistant or non-responder) phenotypes can be clearly defined and the critical environmental exposure of smoking can be measured allowing stratification or matching to be done.

## EPIDEMIOLOGY OF LUNG CANCER

Like COPD, 85–90% of lung cancer can be attributed to smoking exposure (Alberg and Samet, 2003). While smoking is almost a pre-requisite to getting lung cancer, other risk factors are relevant. Age is an important risk factor for lung cancer risk, with risk increasing exponentially after age 60 years old in smokers compared to non-smokers (Woloshin et al., 2008). When age and pack year exposure are compared with reduced FEV_1_ in a multivariate analysis, reduced FEV_1_ (COPD) confers a fivefold increased risk of lung cancer, two- to threefold greater than that conferred by age or pack years alone (Burrows et al., 1977). Indeed prospective studies have shown that COPD predates lung cancer in 65–70% of all cases (de Torres et al., 2007; Wilson et al., 2008; Young et al., 2009b). If computed tomographic (CT)-based emphysema is also assessed, then 85–90% of lung cancer cases have either spirometry-defined COPD and/or CT-based emphysema (de Torres et al., 2007; Wilson et al., 2008). Other risk factors for lung cancer include exposure to occupational dusts (asbestos, coal, and silica), low body mass index (BMI) and low educational level (Tammemagi et al., 2011). While 10–15% of lung cancer cases are “non-smokers,” with a predominance of women, it is not yet clear what role other aero-pollutant exposures play (e.g., passive smoke or cooking smoke). To date, there are too few genetic studies in this group to make any firm conclusions about contributing genes. Another area of uncertainty in lung cancer genetics is the relevance of genes underlying mechanisms defining the histological subtypes of lung cancer. For this reason, lung cancer refers to small cell and non-small cell subtypes secondary to smoking and hereafter is described as a single disease for the purposes of this review although this is unlikely to be the case.

In contrast to COPD, twin studies suggest heritability of lung cancer is only 15–25% suggesting other factors such as smoking history and susceptibility to COPD are of importance (Lichtenstein et al., 2000). Detailed studies of family history however show that having one first degree relative with lung cancer increases your risk by 1.5- to 2-fold that of someone with no family history (Jonsson et al., 2004). While some advocate that family history is a very good marker of “genetic risk” (Valdez et al., 2010), this completely overlooks the over-riding requirement of a smoking history (reducing penetrance of lung cancer disposition in non-smokers) and that a positive family history is reported in only 20% of cases (low sensitivity; Young et al., 2009b). This contrasts with COPD where a positive family history is found in 30–40% of those with COPD (Young et al., 2011b).

With the recent interest in screening for lung cancer using computed tomography of the lung (National Lung Screening Trial Research Team, 2011), there has been growing interest in the role of “COPD” in lung cancer risk (Sekine et al., 2012). Several lung CT screening studies have reported their findings on the relative importance of spirometry-defined FEV_1_ and CT-defined emphysema in lung cancer (de Torres et al., 2007; Mohamed Hoesein et al., 2011; Sekine et al., 2012). While molecular studies implicate both these processes in the development of lung cancer (Young and Hopkins, 2011b), there remains debate about the relative importance of emphysema and reduced FEV_1_ in susceptibility to lung cancer. To date only one study has published results which showed that spirometry-defined COPD and CT-based emphysema overlap in 70% of cases and are independently associated with increasing risk of lung cancer (de Torres et al., 2007). We conclude that susceptibility to “COPD” is the most important marker of a predisposition to lung cancer in smokers beyond that of how much you smoke (Young and Hopkins, 2011c).

## OVERLAPPING GENETIC VARIANTS AND MOLECULAR BIOLOGY

Over the last 4 years many studies have published the results of genome-wide association (GWA) studies of COPD or lung cancer (El Zein et al., 2012). Consistent with the epidemiological studies of the last 40 years, none of the lung cancer GWA studies have considered the possible role of COPD in the associations they have reported (El Zein et al., 2012). Basically, given the close relationship between COPD and lung cancer, it is very possible that some of the associations described in the lung cancer GWA studies are in fact due to associations with COPD (Young et al., 2008, 2010b,c,d; Lambrechts et al., 2010; de Andrade et al., 2011). This possibility arises because COPD is commonly undiagnosed, unless spirometry is routinely done on all study subjects, and that the frequency of COPD in the lung cancer case–control studies is almost certainly at least twofold different between cases and controls. This is because the prevalence of COPD in lung cancer case series is consistently about 65–70% (de Torres et al., 2007; Wilson et al., 2008; Young et al., 2009b) and about 20% in unselected smoking “controls” (Hill et al., 2010). This means that any association reported in a lung cancer case–control study, where COPD has not been routinely measured (and stratified for), might have spuriously attributed their finding to lung cancer when it might actually be related to COPD (Young et al., 2008, 2011b).

We were the first to show this “confounding” effect in 2008, just months after the first gene for lung cancer was reported in the journals “Nature” and “Nature Genetics” (Young et al., 2008). This observation was possible because we compared the allele and genotype frequencies of the most important “lung cancer” SNP in a study that involved three groups of smokers comprising those with COPD, normal lung function (resistant smokers) and lung cancer (sub-phenotyped for COPD by spirometry). By comparing the frequency of the “lung cancer” causing allele of the nicotinic receptor gene across these three groups, we found the disease allele was increased in COPD and in lung cancer with pre-existing COPD. The frequency of this disease allele was only slightly increased in those with lung cancer alone (but not significant). We have gone on to show something very similar for three other GWA genes recently implicated in COPD that are also relevant to lung cancer (Young et al., 2010b,2011c,d). Interestingly, all of the overlapping genes implicated by the GWA studies encode proteins known to be expressed by lung epithelium and involved in various inflammatory pathways (Young and Hopkins, 2011a).

While more studies are needed to confirm these findings, they suggest that the molecular pathways underlying COPD may also be related to lung cancer development. This has important implications in future studies of lung cancer, as it suggests that lung cancer and COPD should be thought of as closely related diseases. To illustrate this point about mistaken association, we have recently published a study of the GSTM1 null genotype and found that it is exclusively related to COPD and not lung cancer as previously reported (Young et al., 2011b). This implies that past lung cancer studies reporting genetic associations might have been mistaken, an error that is relevant to most lung cancer studies published to date. To summarize, there is strong evidence from the GWA studies to suggest that genes underlying COPD and lung cancer overlap and, that the pathways they implicate involve epithelial cell signaling of several inflammatory pathways (Young and Hopkins, 2011b,c; Young et al., 2011a).

In addition to the findings described above, there has been a growing interest in the cell signaling pathways involved in the development of both COPD and lung cancer (Houghton et al., 2008; Young et al., 2009a; Caramori et al., 2010; Yang et al., 2011). To date, it appears that smoking incites an exaggerated or prolonged inflammatory state in the lung, conferred in part by genetic effects and, dominated by neutrophils and macrophages derived from the systemic circulation. These immune cells of the innate system orchestrate a process of epithelial and mesenchymal tissue remodeling where, to varying degrees, the epithelium produces excessive mucus, the airways of the lungs become fibrotic and the alveoli (air sacs) are destroyed. While the exact mechanisms are not yet understood, early evidence suggests these processes result from a microclimate of excessive growth factors and matrix metalloproteinase activity. Such a microclimate has been implicated in the development of many cancers including lung cancer (Young and Hopkins, 2011b). It is possible that the exact pathways underlying COPD and lung cancer, or both, maybe further elucidated by genetic epidemiological studies (El Zein et al., 2012).

## RISK PREDICTION FOR LUNG CANCER BY COMBINING GENETIC VARIANTS

Numerous groups have developed various risk models for lung cancer (Bach et al., 2003; Cassidy et al., 2006; Spitz et al., 2007, 2009; Young et al., 2009c,d) but only two have used SNP-based markers as part of their risk algorithm (Spitz et al., 2009; Young et al., 2009b). The first non-gene model by Bach used only age, pack years, and asbestos exposure to determine risk of lung cancer (Bach et al., 2003). This has only limited predictive utility. The non-gene risk models by Spitz et al. (2007) omitted age as a risk variable while that of Field and colleagues (Cassidy et al., 2006) omitted COPD. The best non-gene-based model to date comes from a large prospective study based on the Prostate, Lung, Colon, and Ovarian Cancer cohort (Tammemagi et al., 2011). In this model, Tammemagi et al. (2011) include age, pack years exposure, current smoking status, COPD, family history, dust exposure, chest x-ray, and BMI. The predictive utility of these models are often reported according to their receiver operator curve (ROC) *c* statistic. While the ROCs of the Bach, Spitz, Field, and Tammemagi models are reported to be 0.69, 0.59–0.65, 0.70, and 0.78–0.84, respectively, comparison of these models is difficult as performance of the models is highly dependent on the populations in which they have been developed (smokers and non-smokers, smokers only, or heavy smokers only; Tammemagi et al., 2011). Similarly, the contribution of the component risk factors, including genetic markers, is dependent on the populations studied and the other variables in the model. What is clearly shown by Tammemagi, is that combining these variables achieves the greatest predictability and highlights how important age, smoking history and COPD are in the model (Tammemagi et al., 2011). In all models, age is one of the most important variables and probably reflects important biological effects from either the cumulative effects of smoking (or other aero-pollutants) and/or the failure of the immune system to cope with this exposure from about aged 40 years when the risk of lung cancer increases in smokers above that of non-smokers or after 60 years old when this risk increases exponentially (Woloshin et al., 2008). While the Spitz model lacks age (Spitz et al., 2007) and the Field model lacks COPD (Cassidy et al., 2006), none of these models have incorporated genetics factors (other than family history).

**FIGURE 2:**
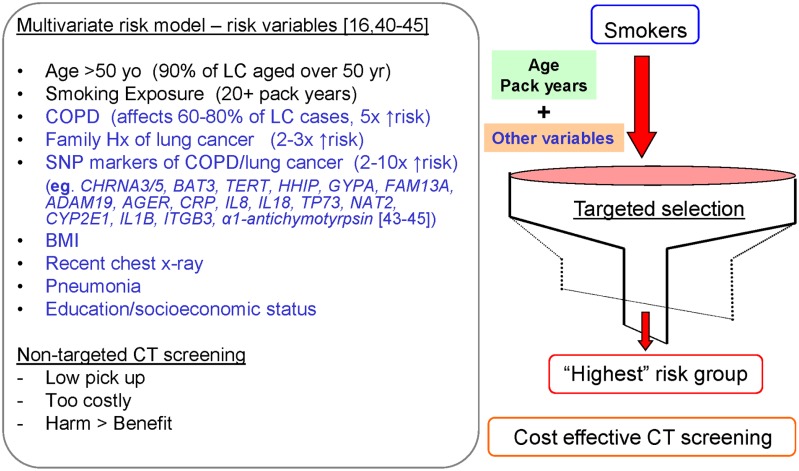
**Multivariate risk model to identify current and former smokers at greatest risk of lung cancer for targeted, cost-effective CT screening**.

We have reported that genetic factors (SNP variants) significantly add to our lung cancer model (Young et al., 2009b,c). This is important as it defines risk in people who are often too young to have a positive family history or who have not yet been diagnosed with COPD. As will be discussed in the next section, this group is important because they are at high risk of getting lung cancer but may miss screening eligibility due to age restrictions (Young and Hopkins, 2012a). Our model includes genes identified by genome-wide studies to be implicated in lung cancer and COPD (**Figure 2**; Young et al., 2011d). This is analogous to including obesity genes in a risk model for diabetes (e.g., FTO – fat-free mass gene) or cholesterol genes in a risk model for heart attack. In a ROC analysis of our gene-based risk model, the *c* statistic ranges from 0.72 to 0.79 with the genetic risk score (from the combined SNP panel) contributing approximately 50% of the total predictive utility, three- to fourfold greater than from family history alone (Young et al., 2009b,c,2011d). Not only does the genetic risk score contribute to the overall score independently of the other variables, for younger smokers, the risk was entirely derived from their SNP genotypes. In contrast to other risk models, our model was developed by comparing heavy smokers with widely disparate outcomes (susceptible vs resistant). Using these cohorts of smokers, we have been the first to show that genes conferring susceptibility to lung cancer involved genes also conferring susceptibility to COPD and vice-versa (Young et al., 2008, 2010b,2011c,d). While this might appear intuitive, of greater importance is that we found some genes that confer a protective effect on COPD also confer a protective effect for lung cancer (Young et al., 2010b,2011c,d). The contribution of these “protective” genes is very important in our risk model as they help to distinguish smokers at least genetic risk and most genetic risk (Young et al., 2009b,c), rather than just those at the greater risk-based solely only susceptibility SNPs (Spitz et al., 2009). What is important in these risk models is that the combination of these variables is superior to the use of only a few variables (e.g., age and smoking) and that genetic factors must be used in combination with the relevant clinical variables to have any useful predictive utility (Young et al., 2009b,c). Of note, we have used susceptible and protective genotypes (rather than alleles) in our risk score, better reflecting the effects conferred by “smoking-sensitive” genes like the α1-anti-trypsin gene (Young et al., 2012a).

An important limitation of many of the risk models described above is that they have been developed in populations including non-smokers or light smokers (Bach et al., 2003; Cassidy et al., 2006; Tammemagi et al., 2011). When these people are included in model development, factors such as smoking history become more important than they would be if only heavy older smokers were involved in model development (Tammemagi et al., 2011). This might dilute out the effect of having COPD or genetic effects (like family history or SNP variants) where smoking exposure dose is critical to gene penetrance (Young and Hopkins, 2011d). The risk of lung cancer in these groups is too low to be included in early diagnosis strategies like CT screening (discussed further below). Lung cancer risk models developed in these wider populations, including low-risk people, might not perform as well in identifying high risk of lung cancer in heavy smokers where CT screening is recommended. Validation in the CT screening studies will be needed to confirm their utility in this setting.

## CLINICAL APPLICATION OF A LUNG CANCER RISK MODEL IN CT SCREENING AND SMOKING CESSATION

Given lung cancer now accounts for between 20 and 30% of all lung cancer deaths yet was rare before 1900 (Young et al., 2012a), it is clear smoking is central to the development of lung cancer. However, genetic make-up is also important in determining which smokers are highly susceptible or resistant to COPD and lung cancer. The genetic factors underlying this predisposition are now better understood thanks to the GWA studies that have identified several genetic loci underlying COPD or lung cancer (Amos et al., 2008; Hung et al., 2008; Thorgeirsson et al., 2008; Broderick et al., 2009; Castaldi et al., 2009; Pillai et al., 2009; Wilk et al., 2009). The good news is both diseases can be all but prevented if smokers quit before age 40 years old (Peto et al., 2000; Anthonisen et al., 2005). Similarly, the progression of impaired lung function to COPD and/or lung cancer (Peto et al., 2000; Anthonisen et al., 2005) can be largely delayed if smokers with this predisposition quit before too much lung damage is done. This means that gene-based risk testing can identify high risk smokers early in their lives, at a time when quitting might prevent the development of these diseases. If our observations are correct, that the pathogenic processes underlying COPD also predispose some smokers to developing lung cancer, then prevention of the former might also prevent development of the latter. In this regard novel chemo-preventive approaches are also possible (Walser et al., 2008; Young et al., 2009a).

In regards to smoking cessation, a recent review concluded that while genetic tests can increase motivation to quit, there is no conclusive evidence that gene-based risk testing (mostly for lung cancer susceptibility), helps smokers quit (Smerecnik et al., 2012). However, this conclusion was based on single marker tests where smokers were assigned as positive or negative and the risk conferred by the test was small. In one underpowered study, those smokers undergoing genetic testing had increased quit rates at both 6 and 12 months (McBride et al., 2002). Sadly data is limited in this regard. We have developed a multivariate gene-based risk test for lung cancer risk that appears to increase smokers uptake of nicotine replacement treatment and improves quit rate (Hopkins et al., 2011, 2012). This observation is the subject of a follow-up study. The important distinction with our test compared to the old single marker tests (Smerecnik et al., 2012) is that no smokers are “negative,” instead all smokers are at some degree of risk of lung cancer and only non-smokers are at low risk. This is important as smokers understand their risk of a smoking-related disease is partly genetically determined and not “negative.” We suggest that such a beneficial effect might occur because gene-based testing helps increase motivational tension and undermine unrealistic optimism (Weinstein et al., 2005; Young et al., 2010a) in many smokers.

The recent publication of the National Lung Screening Trial (NLST) has confirmed that annual lung CT screening of high risk smokers for lung cancer reduces lung cancer mortality by 20% (National Lung Screening Trial Research Team, 2011). However, the eligibility criteria for that study was based on age and pack years only and effectively excludes over 50% of lung cancer cases due to ineligibility for screening (Young and Hopkins, 2012a). Recent recommendations have suggested that the age and pack years criteria for CT screening be widened and that a multivariate approach be used to identify the smokers at greatest risk (Bach et al., 2012; Jaklitsch et al., 2012; Young and Hopkins, 2012b). We believe this is the best way to correctly stratify smokers by risk level and identify those for whom CT screening is most appropriate. From a cost-effective viewpoint, maximizing the detection rate of lung cancer might help make screening more cost-effective (**Figure 2**; Young et al., 2012b). We are currently undertaking a study where our gene-based risk test for lung cancer is being assessed in NLST participants to explore the utility of the test in improving the sensitivity of current screening criteria and increasing lung cancer detection rates.

## SUMMARY

With recent advances in genetic epidemiology, there are a number of genetic loci recently identified that underlie COPD and lung cancer. We have identified that many of these genes are overlapping and can be used in a lung cancer risk model in conjunction with clinical variables. More importantly, this gene-based risk model appears to be effective in engaging smokers in risk mitigating activities (Young and Hopkins, 2012c), a critical utility that non-gene-based risk tools have yet to demonstrate. While further studies are needed to confirm these recent findings, it appears that we are on the verge of being able to use gene-based risk models for assessing lung cancer susceptibility and engage smokers in evidence-based risk mitigating activities.

## Conflict of Interest Statement

The authors declare that some of the studies from their group, cited in this review article, were supported by academic grants and funding fromSynergenz Bio- Science Ltd. Synergenz BioScience holdpatents related to gene-based risk testing in chronic obstructive pulmonary disease and lung cancer.Dr Robert Young is chief scientific advisor to Synergenz Bioscience Ltd. The remaining authors have no conflict of interest.
